# Quantifying the Displacement of Mismatches in Multiple Sequence Alignment Benchmarks

**DOI:** 10.1371/journal.pone.0127431

**Published:** 2015-05-19

**Authors:** Punto Bawono, Arjan van der Velde, Sanne Abeln, Jaap Heringa

**Affiliations:** 1 Centre for Integrative Bioinformatics (IBIVU), VU University Amsterdam, Amsterdam, The Netherlands; 2 Amsterdam Institute for Molecules Medicines and Systems (AIMMS), VU University Amsterdam, Amsterdam, The Netherlands; University of Michigan, UNITED STATES

## Abstract

Multiple Sequence Alignment (MSA) methods are typically benchmarked on sets of reference alignments. The quality of the alignment can then be represented by the sum-of-pairs (SP) or column (CS) scores, which measure the agreement between a reference and corresponding query alignment. Both the SP and CS scores treat mismatches between a query and reference alignment as equally bad, and do not take the separation into account between two amino acids in the query alignment, that should have been matched according to the reference alignment. This is significant since the magnitude of alignment shifts is often of relevance in biological analyses, including homology modeling and MSA refinement/manual alignment editing. In this study we develop a new alignment benchmark scoring scheme, SPdist, that takes the degree of discordance of mismatches into account by measuring the sequence distance between mismatched residue pairs in the query alignment. Using this new score along with the standard SP score, we investigate the discriminatory behavior of the new score by assessing how well six different MSA methods perform with respect to BAliBASE reference alignments. The SP score and the SPdist score yield very similar outcomes when the reference and query alignments are close. However, for more divergent reference alignments the SPdist score is able to distinguish between methods that keep alignments approximately close to the reference and those exhibiting larger shifts. We observed that by using SPdist together with SP scoring we were able to better delineate the alignment quality difference between alternative MSA methods. With a case study we exemplify why it is important, from a biological perspective, to consider the separation of mismatches. The SPdist scoring scheme has been implemented in the VerAlign web server (http://www.ibi.vu.nl/programs/veralignwww/). The code for calculating SPdist score is also available upon request.

## Introduction

Since its inception in the 1970s—early 1980s [1, 2], Multiple Sequence Alignment (MSA) has been one of the most prominent computational techniques in modern molecular biology, and its significance and popularity has only increased since the advent of next-generation sequencing (NGS) techniques. MSA plays a crucial part in various types of biological analyses, e.g. inference of phylogenetic relationships between organisms, conserved sequence elements, functional regions, correlated mutations, and is a vital prerequisite for protein homology modeling [3–9]. MSA methods are used to align multiple homologous sequences, i.e. sequences that are assumed to share a common evolutionary origin; in the resulting alignment the matched residues should indicate that they derive from a single residue in an ancestral sequence, implying that they are structurally superposable and/or involved in a common function [10].

There are two main approaches which are used to assess the quality of a MSA based on sequence information. One is a reference-independent alignment score, that can be calculated solely from sequence information of a single MSA, which is very useful in case curated or structural information on proteins of interest is unavailable. An example of such a score is the sum-of-pairs scoring scheme (which we will refer to as standalone_SP score)[11]. This score is calculated for a stand-alone MSA by exploiting an evolutionary model from which probabilities of pairwise residue conservations and mutations are derived [12, 13]. Such a score is typically used as the objective score for MSA methods, but may also be used to judge the quality of a resulting MSA. The standalone_SP score, calculated using general residue substitution matrices, is rarely used to benchmark MSA methods as the more evolutionary divergent sequence sets will attain lower standalone_SP scores than less divergent sets, making comparison and normalization difficult. Furthermore, an alignment with a high standalone_SP score does not necessarily correspond to a biologically correct alignment as this score does not take structural similarity and conservation of functional residues into account. Therefore its use as benchmark score is not insightful.

The most widely used approach to benchmark alignment quality is referred to as reference-dependent benchmarking, which aims to compute the similarity between the MSA in question with a gold standard reference alignment of the same sequence set. Here, there is no a-priori tendency for more divergent alignments to score lower, although attaining high query-reference alignment similarity is known to be more difficult for evolutionary divergent cases [14].

To address these complications, more recent techniques for assessing alignment quality abounded, which make use of additional information such as protein tertiary structure information. However, since these methods do not rely on sets of reference alignments, they are referred to as reference-independent benchmark techniques [15]. Armougom *et al*. [16] exploited the structural distance between pairs of residues to score pairwise sequence alignment quality without the need for structural superpositioning of the associated tertiary structures. This type of alignment assessment methods are especially useful in pairwise target-template alignment quality assessment, for example in homology modelling and threading algorithms [17, 18].

The main goal of MSA benchmarking is to measure the ability of different MSA methods to reproduce trusted reference alignments [19]. As already mentioned, these reference alignments are normally obtained from a manually (or semi-automatically) curated alignment database, such as BAliBASE [20], OXBENCH [21], PREFAB [22], or SABmark [23]. It has been shown that in addition to the divergence of the sequences, the performance of an MSA method depends also on several other factors such as sequence lengths, the presence or absence of large insertions and N/C terminal extensions, or over-representation of one or more protein subfamilies [14]. Therefore, a thorough comparative analysis of MSA methods requires accurate reference alignments representing various alignment problems to objectively assess the quality of MSA methods. The ultimate benefit of MSA benchmarking to the user is knowing what is the best method for a specific alignment problem. Therefore, the availability of scoring schemes that bring out different strengths and weaknesses of an alignment method is important.

**Fig 1:**
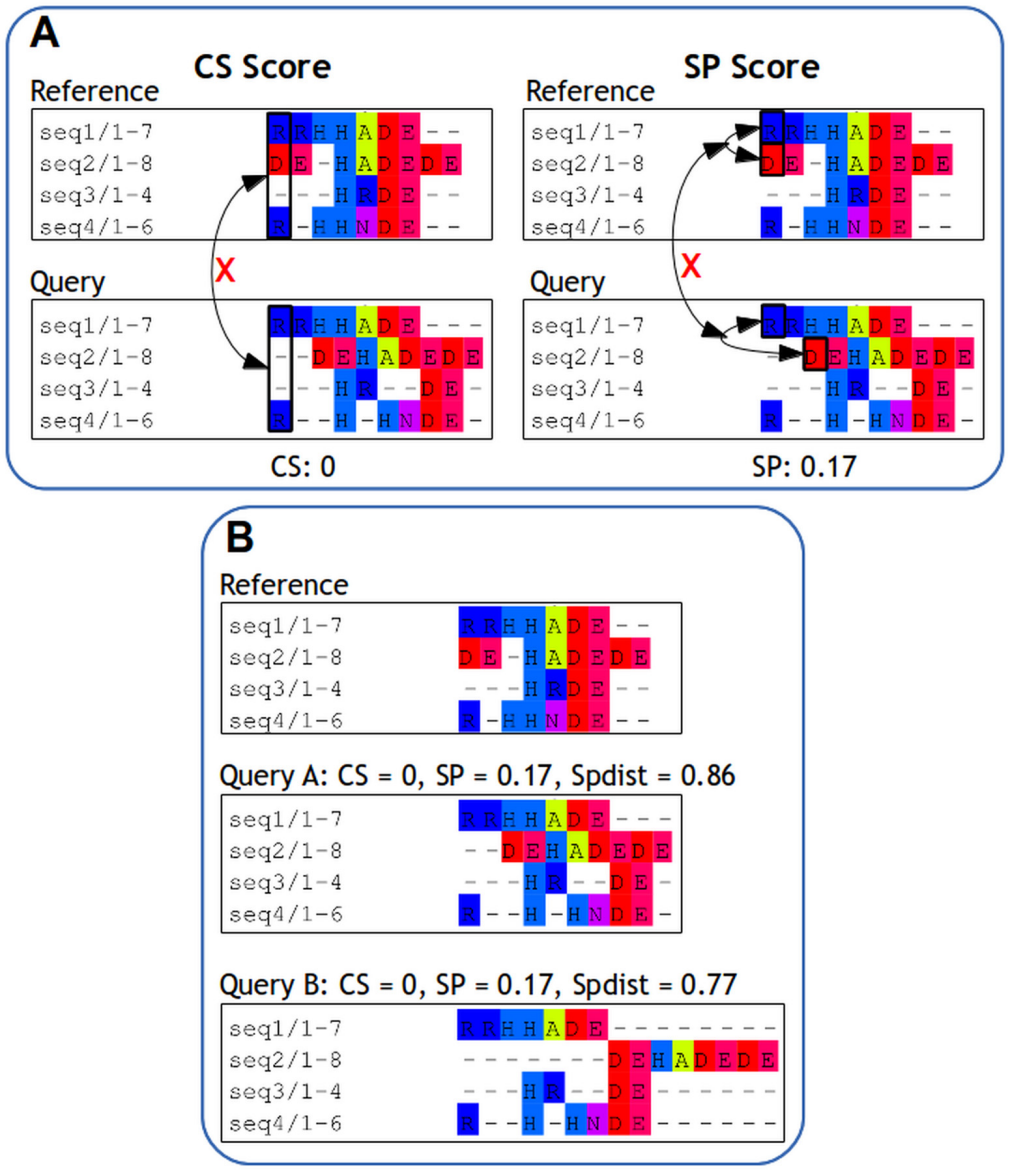
(A): CS score compares columns in the reference to corresponding columns in the query; SP score compares all aligned pairs in the reference to the corresponding pairs in the query. (B): Toy alignments to illustrate the difference punishment for alignment shifts in CS, SP, and SPdist (Eq 8) scores. All sequences in this contain a small sequence motif H-x-D-E which has to be aligned correctly. Both query A and B contain the same number of mismatches (indicated by the same SP score for both queries), the sole difference between these two queries is that sequence 2 in query B is shifted five positions further. In this situation one might argue that although both alignment are wrong, i.e. the sequence motifs are not aligned properly, query A is a slightly better alignment than B as the motifs in query A is positioned closer to each other than in query B.

The similarity between a reference alignment and the corresponding query alignment is commonly calculated using the column score (CS) and the sum-of-pairs (SP) score. The CS score calculates the query-reference alignment similarity as the fraction of columns in the reference alignment that is exactly reproduced in the query alignment, meaning that a single mistake in a MSA column (i.e. a mismatch in a single sequence) will disqualify the entire column (Fig 1A). The SP score determines the similarity by the fraction of aligned residue pairs in the reference alignment that is correctly reproduced in the query alignment (Fig 1A), i.e. the proportion of mistakes over all residue pairs in a column is reflected in the score. As a result, the SP score is softer and less strict than the CS score. Note that this type of SP score is not the same as the standalone_SP score as it takes the reference alignment and not the amino acid substitution matrix into account.

Fig 1 illustrates the difference between CS and SP scores and their limitations. In the toy example (Fig 1B) one can observe that the CS score plummets to 0 due to minute shifts in the sequences. This indicates that the CS score is highly influenced by the number of the sequences and the gap locations compared to the SP score. The toy example also shows that query alignment A is more similar to the reference alignment than query alignment B, hence query alignment A should have a better score than query alignment B. However, this is not the case if the similarity between the query and the reference alignments are to be measured with the CS and SP score. Since both scoring schemes only take matches and mismatches into account, they are not able to discriminate whether one mismatch, i.e. a misplaced residue, is more shifted than the other.

Due to their nature, SP and CS scores may not be discriminative for MSAs that contain long stretches of gaps. For example, one MSA method may detect a long insertion/deletion appropriately, but get the boundaries slightly wrong, resulting in a small shift, while another method may miss the insertion/deletion altogether. The former case is arguably less incorrect, but may yield similar SP and CS scores as the latter (see the case study in the “Results and Discussion” section for a real-life example of a similar situation in MSA benchmark). The key problem is that the SP and CS scores do not take the severity of displacement of mismatches into account. In practice the length of misalignments often plays a major role in biological analyses where the accuracy of an MSA is of paramount importance, for example in analyses where manual alignment editing is required to achieve a biologically sound alignment. In such cases, misalignment of (stretches of) identical or similar residue pairs with relatively small shifts will be much easier detected by the human eye than misplaced residues positioned further away from their correct position in the alignment.

The purpose of this study is to investigate whether one can obtain a more fine-grained indication of how much a query alignment actually differs from the reference by taking distances between mismatched residue pairs into account in MSA comparison, which is particularly meaningful in the case of alignments with long stretches of insertions/deletions. In order to do this we developed a variant of the SP score, SPdist, which takes into account the distance of mismatched residue pairs instead of the binary classification into match and mismatch state as in the case of the standard SP score. Cline et al. (2001) devised a distance-based scoring scheme for pairwise alignments called shift score which also aims to differentiate the treatment between major and minor shifts in alignment comparison [24]. In this study we will also compare the behaviour of our SPdist score with the shift score.

## Methods

### MSA benchmark data set

BAliBASE is one of the most widely used alignment database to assess the quality of MSA methods. It contains various MSAs obtained from manually curated 3D structural superpositions [20]. The alignments in BAliBASE are organized in reference sets, each representing a specific alignment problem: Reference 1 (RV11 and RV12) contains alignments of equidistant sequences; Reference 2 (RV20) holds alignments of protein families with with one or more so-called orphan sequences(< 25% identical); Reference 3 (RV30) comprises alignments of divergent protein subfamilies with < 25% sequence similarity between subfamilies; Reference 4 (RV40) has alignments of sequences with large N/C terminal extensions; Reference 5 (RV50) alignments with large internal insertions; while Reference 6–8 contain highly specialized alignment problems such as, transmembrane proteins, proteins with multiple repeats, and inverted domains. Each reference set in BAliBASE contains two types of alignments: a full-length alignment and a truncated one containing only highly conserved regions. We selected the full-length alignments for this study as they are more akin to real alignment situations. Out of the above eight reference sets in BAliBASE, we only selected sets 1 to 5 for our analysis since the remaining sets represent more specific alignment situations that are less amenable to the generic MSA methods assessed in the current study. The five selected benchmark sets contain 218 reference alignments in total. Although the aforementioned reference alignment databases PREFAB [22] and SABmark [23] are much larger than BAliBASE, both these datasets only provide pairwise alignments and no true MSA as reference for each alignment case, which is undesirable for the SPdist score introduced in this paper. Similarly, the smaller OXBENCH reference set [21] comprises multiple sequence alignments containing regions that are annotated as unreliably aligned, which also makes this database less amenable to evaluating the SPdist score.

### Generation of multiple sequence alignments

Various multiple sequence alignment programs were selected for this study, namely ClustalW2 (ver. 2.0.10) [25], Clustal Omega (ver. 1.2.0) [26], MAFFT (ver. 7.215) [27], Muscle (ver. 3.7) [22], ProbCons (ver 1.12) [28], and Praline [29]. As the aim of this study is to analyse the behavior of the SP and SPdist scoring schemes, and not to select the best alignment program, we made no attempt to optimize these alignments programs and ran them using their default settings for all test sets. This holds particularly for the Praline method, where, out of a number of different alignment strategies, the suboptimal standard progressive alignment technique was used as a control. An exception was made for MAFFT, which was run using both the default and optimized parameter settings.

### SP and CS scoring schemes

**Fig 2:**
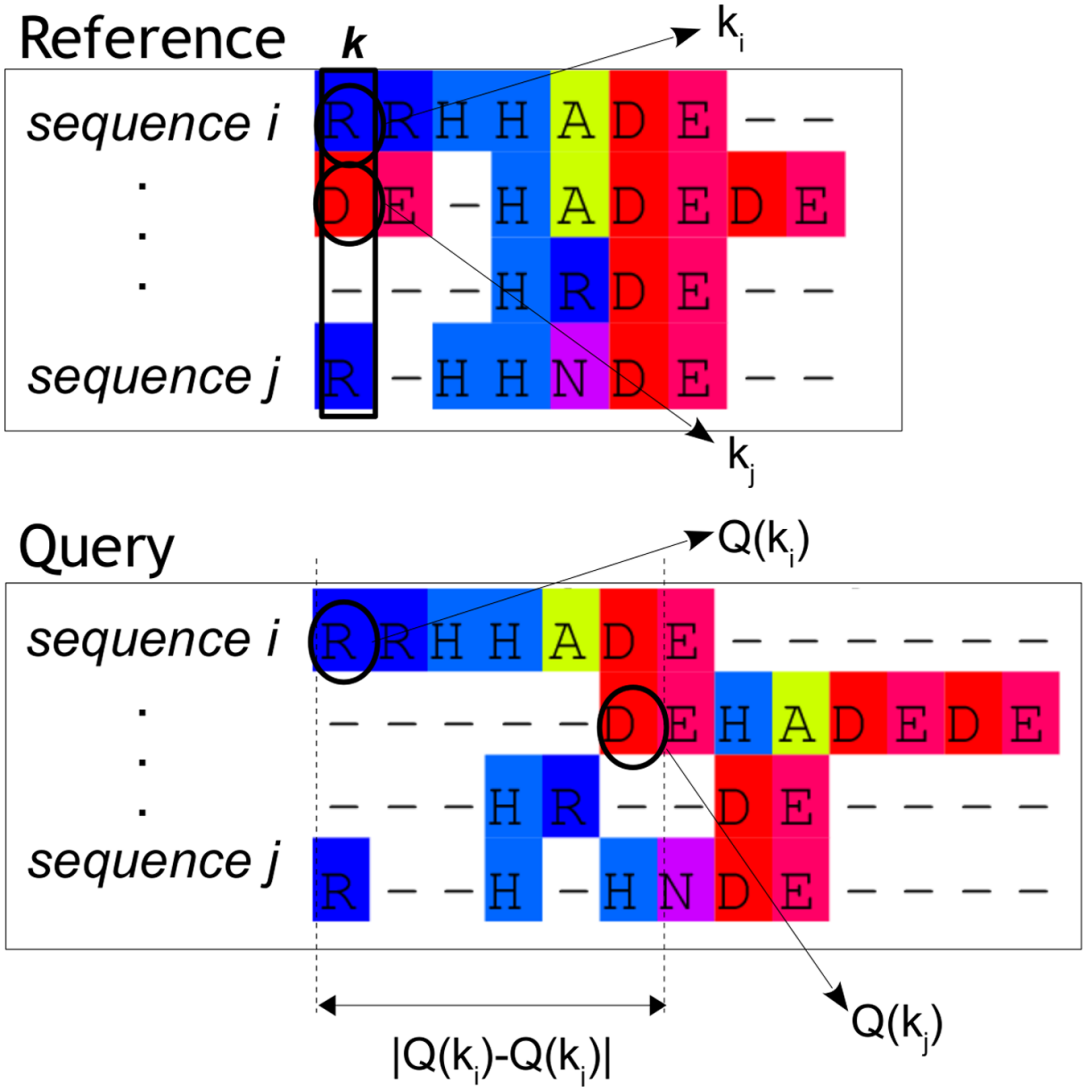
Illustration of the mapping of aligned residue pairs *k*_*i*_ and *k*_*j*_ in reference alignment onto the corresponding pairs in the query alignment (*Q*(*k*_*i*_) and *Q*(*k*_*j*_)). The distance for residue pair *k*_*i*_ and *k*_*j*_ is calculated as the absolute difference in alignment columns between *Q*(*k*_*i*_) and *Q*(*k*_*j*_).

The column score (CS) is calculated as the percentage of correctly aligned columns for a reference and query alignment, having *M* sequences and *N* columns in the reference alignment:

$$\begin{array}{c} CS=\frac{\sum_{k=1}^{N}compare\left(k\right)}{N} \end{array}$$ (1)

$$\begin{array}{c} compare\left(k\right)=\{\begin{array}{cc} 1 & \;\text{if}\;Q(k_{1})=Q(k_{2})=\ldots =Q(k_{M})\\ 0 & \;\text{otherwise} \end{array} \end{array}$$ (2)

where *Q*(*k*_*i*_) is a lookup function that translates the column of a residue *k*_*i*_ of sequence *i* in the reference alignment into the corresponding column in the query alignment (Fig 2). The *compare* function checks if the positions of the residues in a column *k* of the query alignment correspond to a single column in the reference alignment. If all residues compared are in the same column, the result is 1, and 0 otherwise. For all columns in the reference alignment the positions of the residues in the query alignment are compared. The outcomes over all columns are then summed and divided by the total number of columns in the reference alignment. The sum-of-pairs score (SP) is calculated by taking all aligned pairs of residues in the reference and the corresponding pairs in the query alignment into account:

$$\begin{array}{cc} SP & =\frac{\sum_{k=1}^{N}\sum_{i=1}^{M}\sum_{j=i+1}^{M}match(k_{i},k_{j})}{\frac{1}{2}NM(M-1)} \end{array}$$ (3)

$$\begin{array}{cc} match(k_{i},k_{j}) & =\{\begin{array}{cc} 1 & \;\text{if}\;Q(k_{i})=Q(k_{j})\\ 0 & \;\text{otherwise} \end{array} \end{array}$$ (4)

Here, each aligned pair *k*_*i*_ and *k*_*j*_ in the reference alignment is compared to the corresponding pair *Q*(*k*_*i*_) and *Q*(*k*_*j*_) in the query alignment. If the pair is indeed also aligned in the query alignment a 1 is added; a 0 otherwise. The total score over all pairwise comparisons is divided by the total number of matched residue pairs $\frac{1}{2}NM\left(M-1\right)$ in the reference alignment.

### Distance-based SP score (SPdist)

In this work we develop a new score, the *SPdist* score, that is similar to the SP score but incorporates distance information between misaligned residue pairs rather than a score of 1 for matches and 0 for mismatches. For each aligned pair of residues in the reference alignment, now the distance between the corresponding two residues in the query alignment is determined. As the final SPdist score, the average mismatch distance over all aligned pairs in the reference alignment is calculated.

The way mismatch distances are incorporated in the SPdist score is inspired by similar measures used in the field of structural alignment, such as the distance-based scoring scheme employed in the ProSup structural alignment method [30] and the Global distance test (GDT_TS) score which is mainly employed in assessing structural similarity between alternative 3D structures of a single protein, where one structure represents the template and the other the model [31]. The GDT_TS scoring scheme determines the number of template-model C*α* atom pairs (*G*(*v*)) that are within the range of a predetermined distance cut-off, *v*. The score is then calculated as the average percentage of residues whose template-model C*α* atom pairs fit within four predetermined distance cut-offs: 1, 2, 4, or 8 Å [32]; as shown in the following formula:

$$\begin{array}{c} GDT\_TS\left(\%\right)=\frac{1}{4}\sum_{v=1,2,4,8}^{}\frac{G(v)}{t}X100 \end{array}$$ (5)

where *t* is number of all C*α* atoms in the template structure. In SPdist we similarly employ binning as a pre-processing method to be able to weight the effects of various alignment shift distances; for example, large shifts would otherwise tend to dominate the final score. SPdist scoring works as follows: First, we calculate the distance between all query residue pairs that are matched in the reference alignment. Then, these residue pair distances are grouped into separate bins, based on distance thresholds *t*, followed by *SPdist*(*t*) calculation over all the thresholds (Eqs 6 and 7)

$$\begin{array}{c} SPdist\left(t\right)=\frac{\sum_{k=1}^{N}\sum_{i=1}^{M}\sum_{j=i+1}^{M}D_{t}\left(k_{i},k_{j}\right)}{\frac{1}{2}NM(M-1)} \end{array}$$ (6)

$$\begin{array}{c} D_{t}(k_{i},k_{j})=\{\begin{array}{cc} 1 & \;\text{if}\;|Q(k_{i})-Q(k_{j})|<t\\ 0 & \;\text{otherwise} \end{array} \end{array}$$ (7)

where *D*_*t*_ is number of residue pairs whose distance is less than *t*. Note that within a bin, a mismatch shift that is smaller than the threshold specified for that bin is counted positively, such that a higher SPdist score for that particular bin implies less mismatch displacement and hence higher similarity between reference and query. Since the scores are cumulative over the bins, i.e. each next bin (for larger alignment shifts) will always contain the SPdist score of the preceding bin, the bin scores grow uniformly. Both the SPdist score for the individual thresholds and the overall average score are scaled between 0 and 1, and can thus be interpreted similarly to the classical SP score.

A single weighted average of SPdist score over all selected distance thresholds can be calculated as follows:

$$\begin{array}{c} avg\_SPdist=\frac{\sum_{t=1,4,10,22,46}^{}W_{t}*SPdist\left(t\right)}{\sum_{t}W_{t}} \end{array}$$ (8)

Where *W*_*t*_ is the weight of threshold *t*. The SPdist scoring scheme allows the user to specify different weights for the different thresholds; for example, the weights of the distance thresholds can be set based on expected frequencies gleaned from a specific collection of alignments. In this study we employed five thresholds (*t* = 1, 4, 10, 22, 46) with equal weights (*W*_*t*_ = 1) to calculate the avg_SPdist score. The thresholds selected are loosely based upon the lengths of secondary and super-secondary structures [33–35], so that the severity of mismatch punishment is calibrated according to misalignment of these structural features. Note that *SPdist*(1) ≡ *SP*.

### Shift score

The shift score [24] is somewhat akin to the SPdist score as both aim to take the displacement of mismatches into account in alignment comparison. However, these scoring schemes differ greatly in the way the score is normalized. For a pair of sequences *X* and *Y* aligned in query alignment *Q* and in reference alignment *R*, the shift score is calculated as follows:

$$\begin{array}{c} SS_{R}\left(Q\right)=\frac{\sum_{i=1}^{\left|Q\right|}CS_{R}\left(Q_{i}\right)}{\left|Q|+|R\right|} \end{array}$$ (9)

$$\begin{array}{c} CS_{R}(Q_{i})=\{\begin{array}{cc} s\left(X_{j}\right)+s\left(Y_{k}\right) & \;\text{if}\;\text{residue}\;\text{pair}\;X_{j}\;\text{and}\;Y_{k}\;\text{are}\;\text{aligned}\;\text{in}\;\text{position}\;Q_{i}\\ 0 & \;\text{otherwise} \end{array} \end{array}$$ (10)

$$\begin{array}{c} S(a_{i})=\{\begin{array}{cc} \frac{1+\epsilon }{1+|shift(a_{i})|}-\epsilon & \;\text{if}\;shift(a_{i})\;\text{is}\;\text{defined}\\ 0 & \;\text{otherwise} \end{array} \end{array}$$ (11)

where |*R*| is number of aligned residue pairs in alignment *R*, *CS*_*R*_(*Q*_*i*_) is the score for position *i* in alignment *R*, *S*(*a*_*i*_) is the score for residue *a*_*i*_ with respect to the reference alignment, and *shift*(*a*_*i*_) is the measure of how much residue *a*_*i*_ is shifted. if residue *a*_*i*_ is not aligned to another residue in either query or reference alignment then *shift*(*a*_*i*_) is undefined.

The shift score ranges from −*ϵ* to 1.0, where *ϵ* is a small number (usually set by default to 0.2) used to differentiate minor shifts from major ones. If two alignments are completely identical, their shift score is 1.0. In this study we employed the shift score as it is implemented in qscore package (http://www.drive5.com/qscore/), because the original web server for calculating the shift score is unfortunately no longer available.

## Results and Discussion

### The behaviour of SP and SPdist scores in MSA benchmark

In order to understand the effect of the severity of mismatch shifts on the alignment score, we benchmarked six different MSA methods against BAliBASE reference alignments using the standard SP score, shift score and the new SPdist score. As our goal is solely to observe the behavior of SP, SPdist, and shift scores, all of the MSA methods that we used were ran using their default settings.

**Fig 3:**
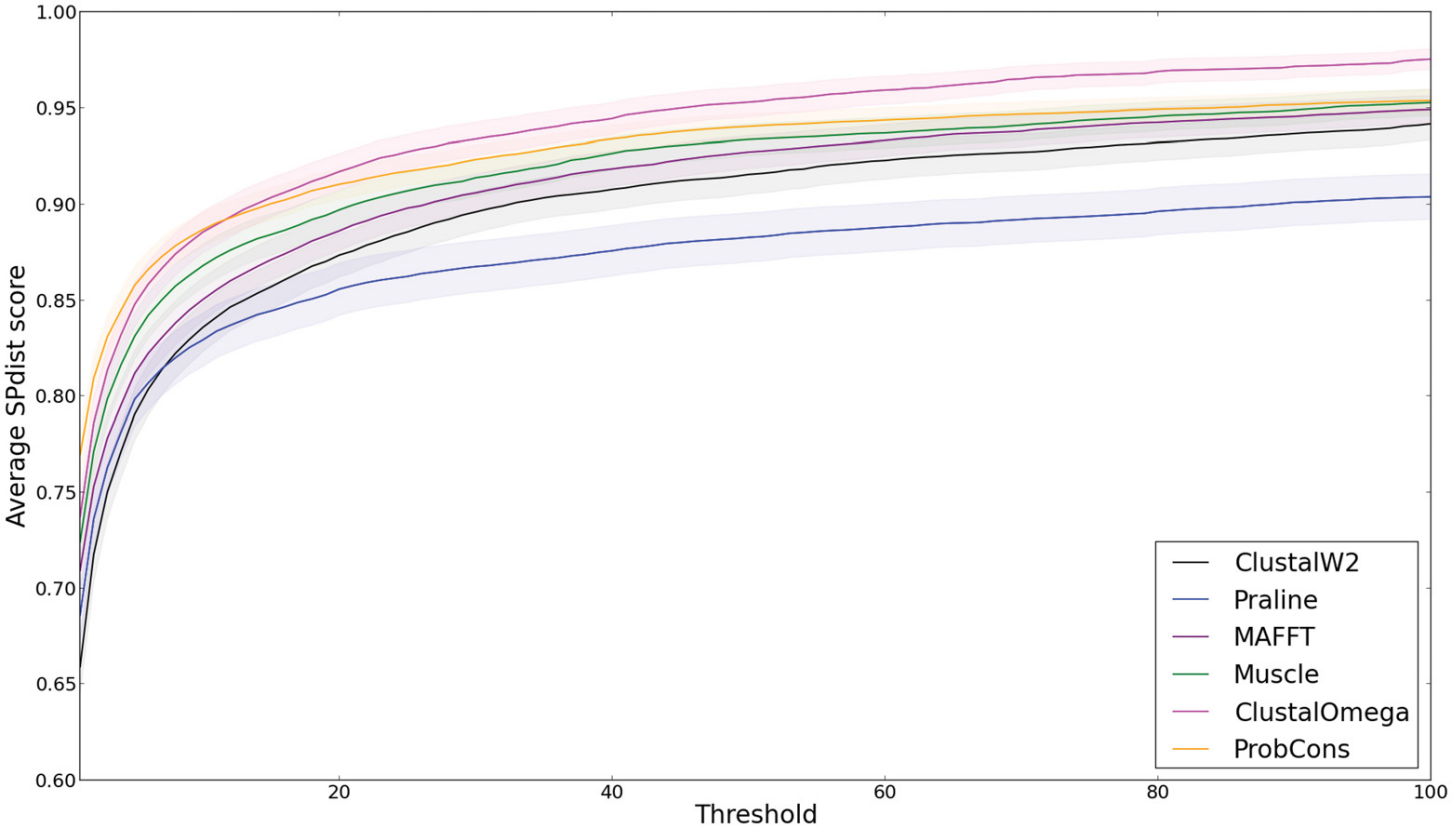
The average SPdist(threshold) for each method against the distance thresholds. The shaded areas represent standard error ($SE=\frac{\sigma }{\sqrt{N}}$). Here the reference sets 1–5 of the BAliBASE dataset are used to calculate the SPdist score. Some methods are more accurate at small distance thresholds, while others converge better at large distances.

We compiled the scores produced by the three scoring schemes to analyse whether the performance rankings produced by these scoring schemes are similar to each other. We calculated the SPdist score (Eq 6) for a large range of distance thresholds (i.e. 1–100), to probe the behaviour of alignment methods as the distance threshold is varied (Fig 3). From this figure it becomes apparent that the MSA methods which obtain the best SPdist scores over small thresholds are not guaranteed to have the best SPdist scores over large thresholds. Hence it is important to consider mismatches using different distance thresholds to evaluate the performance of different MSA methods.

**Fig 4:**
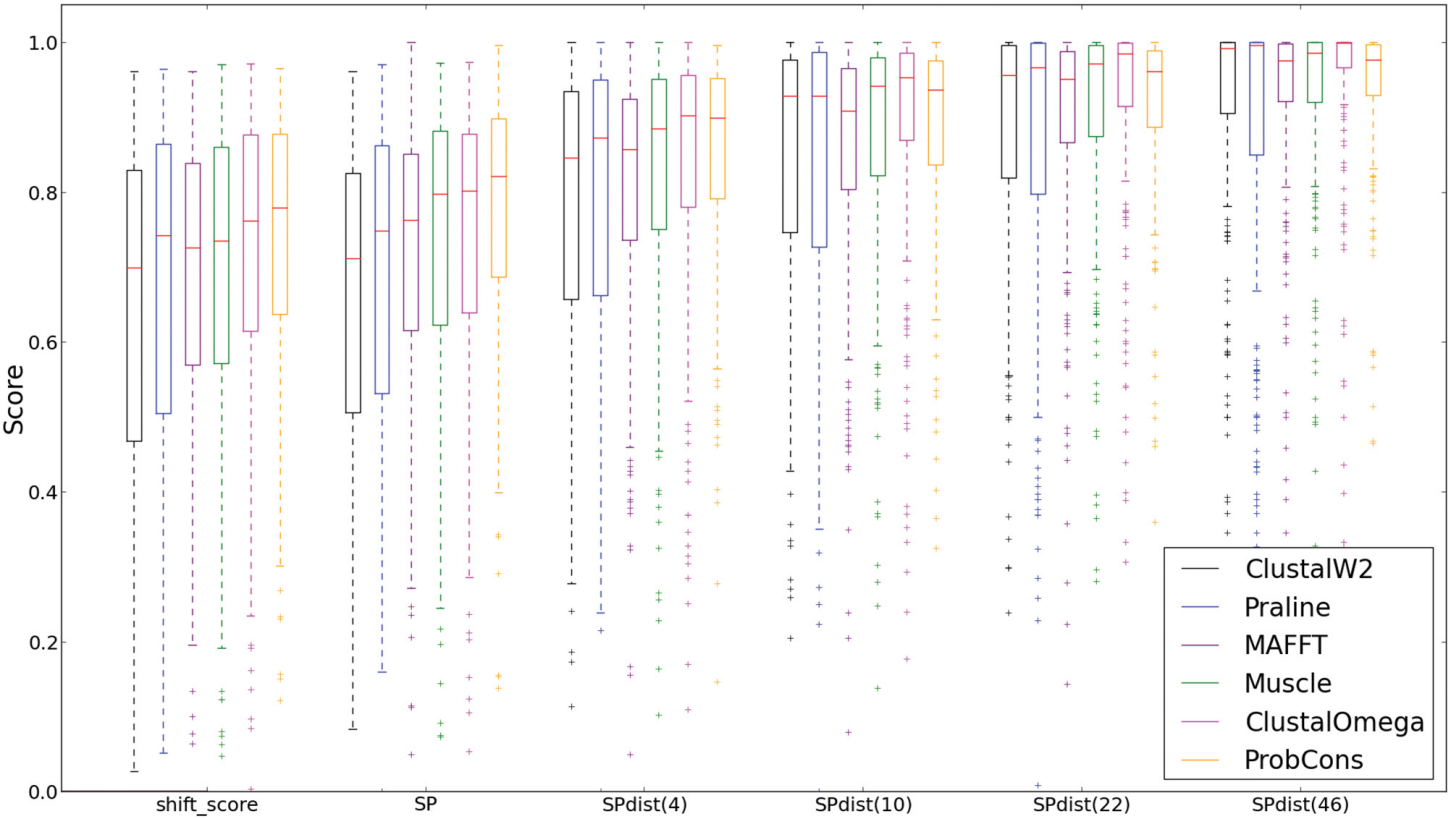
The distribution of the shift score, SP score and the SPdist score. Here the reference sets 1–5 of the BAliBASE dataset are used to calculate the scores. The ranking for the different methods changes at larger distance thresholds, compared to both the SP and the shift score; note that SP score = SPdist(1). Note that the variance of individual alignment score remain high at large distance thresholds. The average values associated with the median values displayed in this figure are reproduced in S1 Table.

Fig 4 shows the distribution of the benchmark scores of the different alignment methods; note that the SP score is identical to SPdist(1). The figure shows that the SP and shift scores produce very similar rankings while the SPdist scoring scheme shows fluctuations in the ranking of the MSA methods across different distance thresholds. The SPdist scores for the various mismatch distance thresholds reveal that the different MSA methods differ in their tendency to insert smaller or larger mismatch shifts.

**Fig 5:**
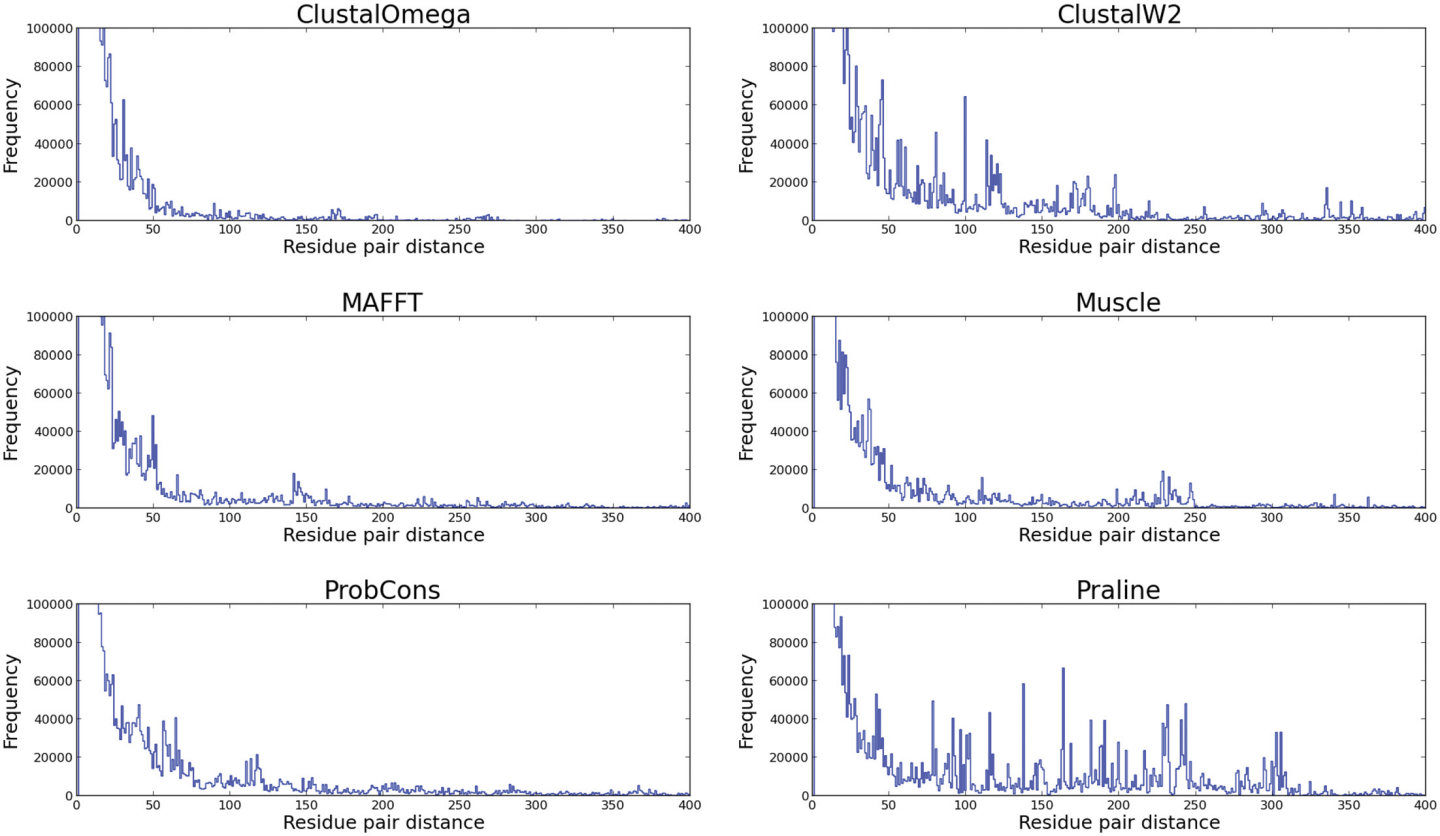
Distributions of residue pairs distances. In this figure one can clearly observe that the tools which perform less well in our benchmark have relatively high number of peaks in the residue pair distances around 50–350s.

For example, one can observe in Figs 3 and 4 that ProbCons alignments contain few small mismatches as it has the best SP/SPdist(1) score; yet at higher distance thresholds the scores drop relative to the other methods. The distribution of the number of gaps in the alignments (Fig 5) shows clearly that overall less accurate methods (ClustalW2 and Praline) tend to generate alignments with longer mismatches.

Moreover, it is striking that for quite a large number of reference alignment sets the MSAs produced by the methods never converge at large distances (Fig 4) which is apparent from the spread of individual scores at larger distance thresholds. This shows that for all methods considered here, there is room for improvement by making these more accurate over longer distances.

The MSA benchmark scores described in this study are not true metrics because they violate the principle of symmetry as the similarity between the reference and query MSAs are based on the fraction of matched residues in the reference MSA that are correctly reproduced in the query alignment; therefore *d*(*a*, *b*) ≠ *d*(*b*, *a*). Consequently, the scores cannot be used to directly compare benchmark results obtained from different datasets [36].

Lastly, we wanted to analyse the relationship between SP and SPdist scoring schemes in relation to the difficulty reproducing the reference alignments. Here, we relate difficulty to the extent of gap insertion in the reference alignments, since alignments with many gaps will more easily accommodate mismatches with larger shifts. We accordingly define “gapness” as a relative measure of the number of gaps in a MSA with respect to the minimum and maximum number of possible gaps in the pairwise sequence alignments underlying the MSA (Eq 12). The rationale behind the gapness measure is to normalize the severity of misplacement according to what can be expected in each of the alignments. Gapness is defined as follows:

$$\begin{array}{c} “gapness”=\frac{\sum_{i,j}^{}\frac{X_{ij}-min_{ij}}{max_{ij}-min_{ij}}}{M(M-1)/2} \end{array}$$ (12)

where *M* is the number of sequences, *Xij* is the number of gaps observed in sequence *i* and *j*, *max*_*ij*_ is the maximum number of gaps possible in the pairwise alignment between sequence *i* and *j* (*max*_*ij*_ = length seq. *i* + length seq. *j*). While *min*_*ij*_ is the minimum number of gaps possible in the pairwise alignment between sequence *i* and *j* (*min*_*ij*_ = |length seq. *i*—length seq. *j*|).

**Fig 6:**
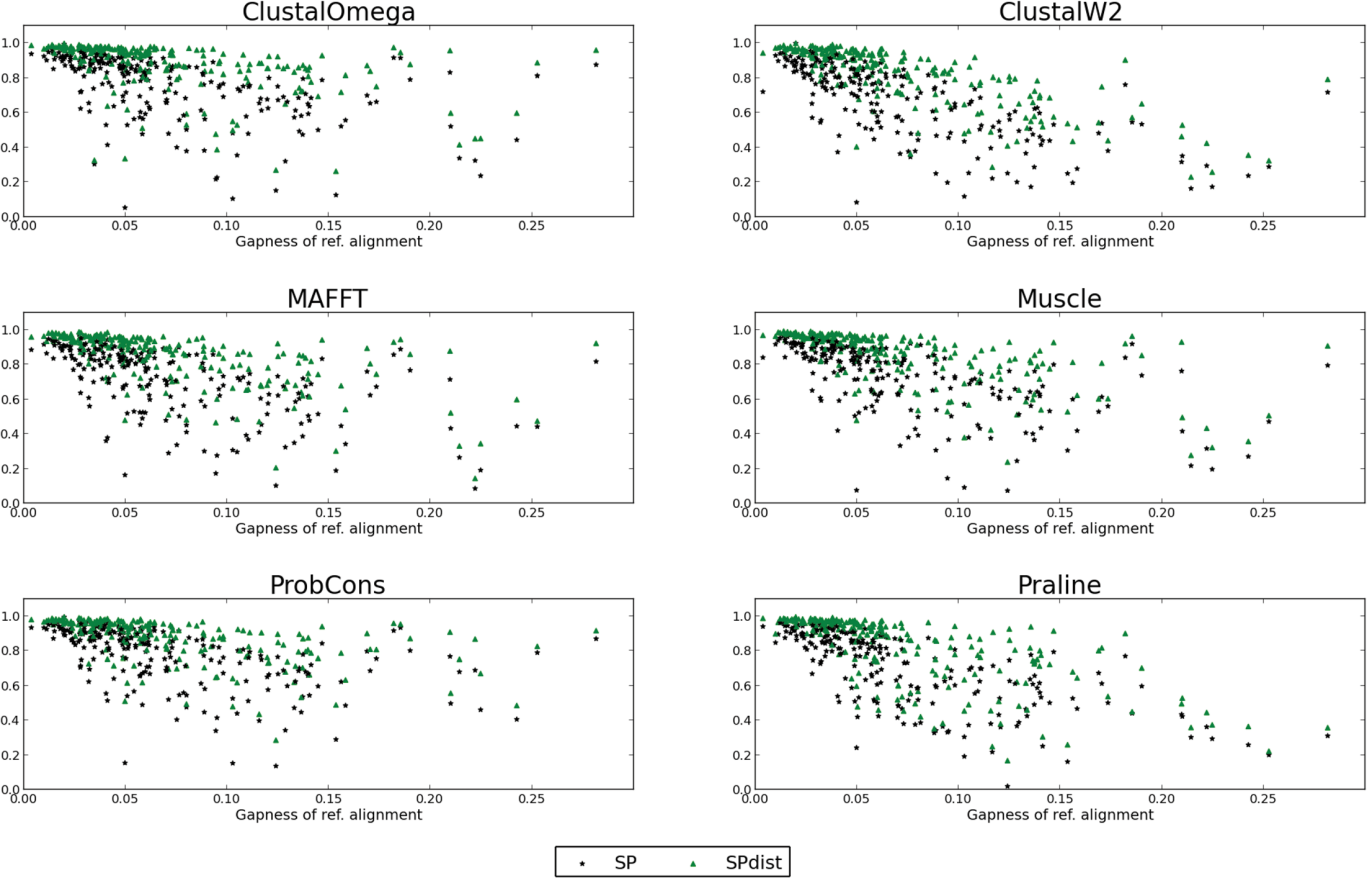
SPdist and SP versus the difficulty of the reference alignment which is expressed as “gapness” scores (g). Both SP and SPdist scores show negative correlation with respect to the “gapness” score. The Spearman’s rhos are -0.69 for SP and -0.72 for SPdist. The Spearman’s rho of SP score and “gapness” score for each method: ClustalOmega = -0.63, ClustalW2 = -0.76, MAFFT = -0.67, Muscle = -0.71, ProbCons = -0.65, and Praline = -0.78. The Spearman’s rho of SPdist score and “gapness” score for each method: ClustalOmega = -0.64, ClustalW2 = -0.80, MAFFT = -0.73, Muscle = -0.75, ProbCons = -0.68, and Praline = -0.78.

Fig 6 shows the comparison of SP and the avg_SPdist in terms of “gapness”. We analysed the correlation between avg_SPdist and SP score with the “gapness” score of the reference alignments by calculating the Spearman’s rank correlation. Both scoring schemes show negative correlation with respect to the “gapness” score, with the avg_SPdist score having a slightly stronger (Spearman’s rho = -0.72) anti-correlation than the SP score (Spearman’s rho = -0.69). Although the gapness score is not measuring amino acid relatedness, it is related to the difficulty of reproducing reference alignments as reflected in the negative correlation with the SP score. As expected, the SPdist scoring scheme is more sensitive to reference alignments containing a larger proportion of gaps than the SP scoring scheme.

### Web server

**Fig 7:**
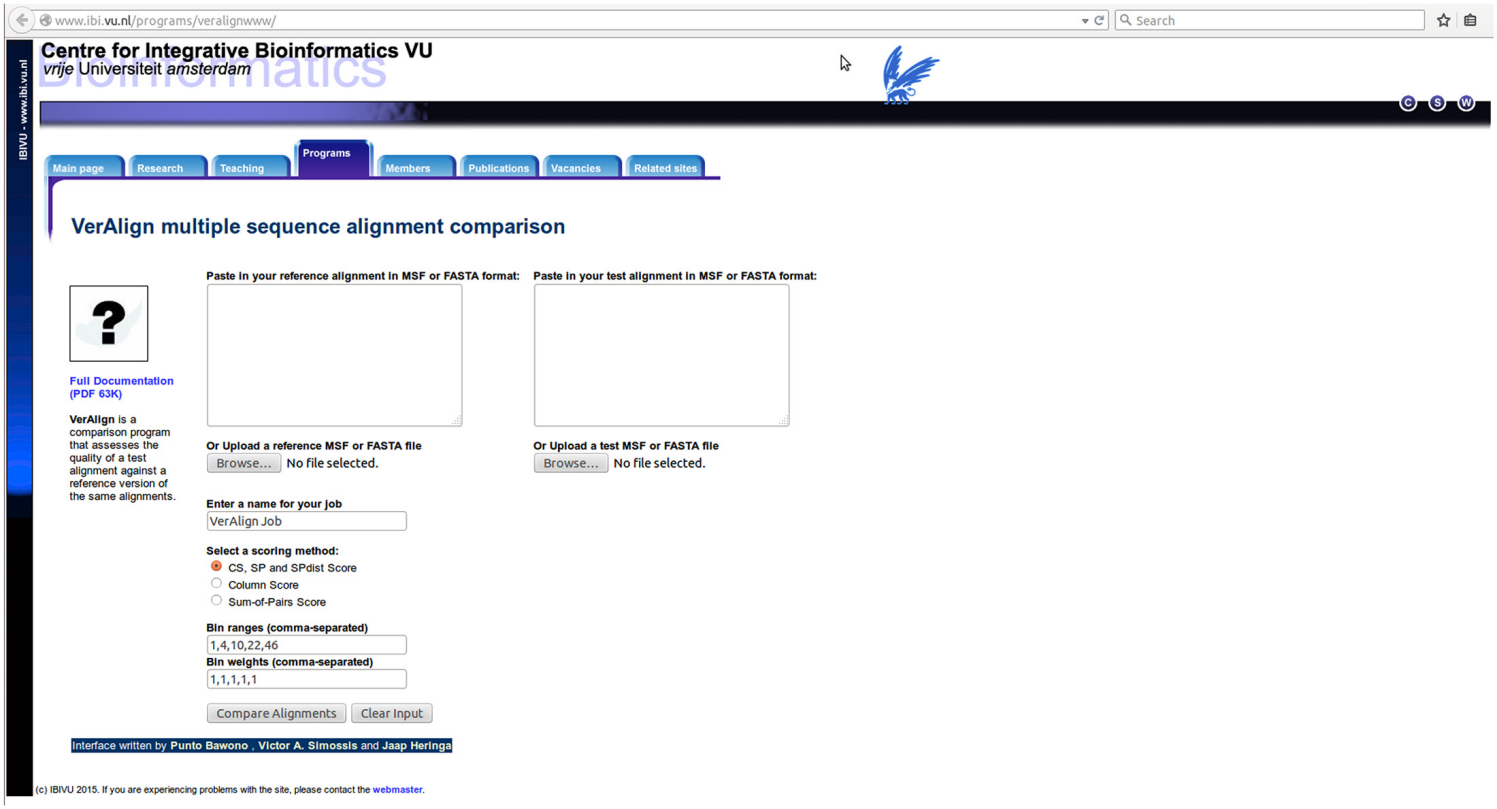
The VerAlign server user interface.

VerAlign is a web tool for calculating the similarity between two MSAs according to the SP, CS, and SPdist scores. This web tool is accessible on the IBIVU website at VU University Amsterdam (http://www.ibi.vu.nl/programs/veralignwww/). VerAlign provides the user with a compact and intuitive user interface. The alignments to be compared must be in fasta or MSF format and can either be entered manually in the text boxes provided or uploaded as files (Fig 7). The user can specify the number of thresholds as well as the associated threshold values and weights via the user interface. Once a VerAlign job is submitted, the user is presented with a holding page that refreshes automatically and displays the results page when the job is completed. The output page displays SP, CS and the SPdist scores of the query alignment. The SPdist scores of the various distance thresholds are displayed alongside the avg_SPdist score. This allows the user to tune an alignment method on the behaviour of mismatch shifts over specified distance thresholds.

**Fig 8:**
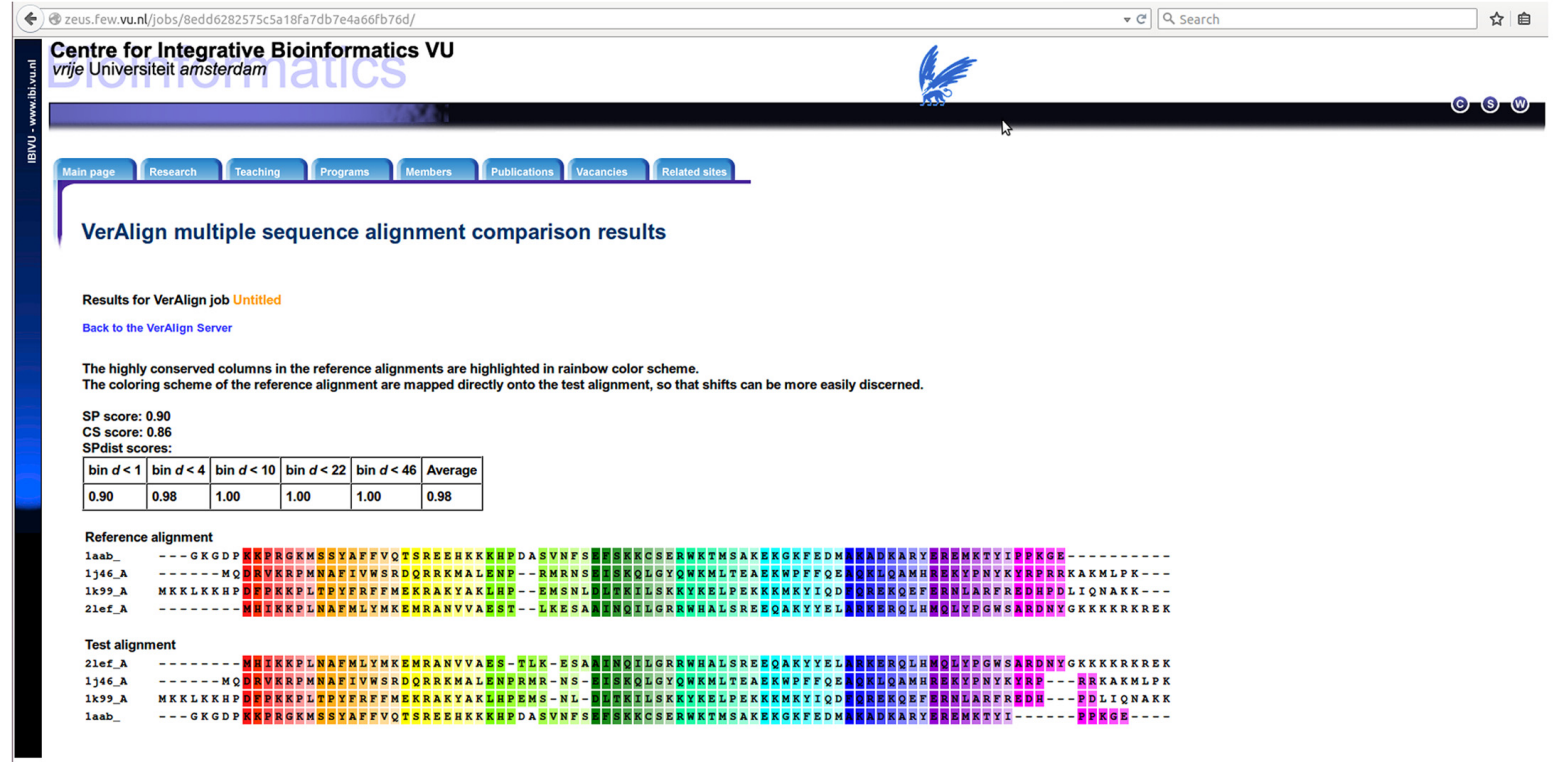
The result page of the VerAlign web server.

Fig 8 shows an example of the VerAlign result page, showing the reference and query alignments and their similarity scores. The columns in the reference alignment that are regarded as highly conserved (i.e. containing 20% or less gaps) are highlighted using a rainbow colour scheme. The colouring scheme of the reference alignment is mapped directly onto the query alignment so that the user can easily track the difference between reference and query alignments.

### Case study

**Fig 9:**
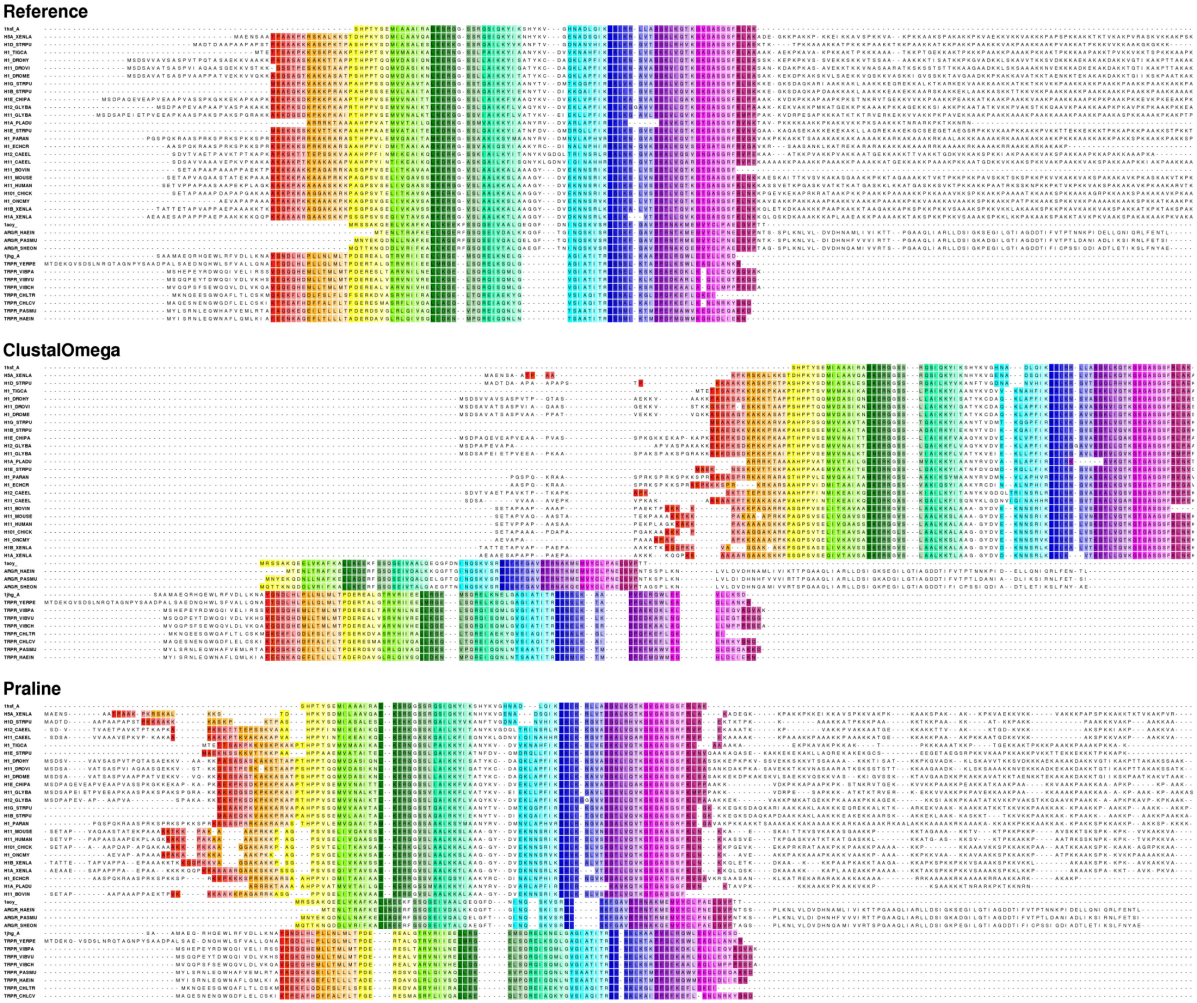
The BAliBASE Reference alignment and query alignments by Clustal Omega and Praline of the sequence set BB30009. For the sake of visibility only core blocks of the MSAs and their flanking regions are shown in this figure. The core blocks in the reference alignment are highlighted in rainbow color scheme. The coloring scheme of the reference alignment are mapped directly onto the query alignments, so that shifts can be recognized easily. The MSA generated by Clustal Omega yielded an SP score of 0.48, shift score of 0.28, and SPdist a score of 0.59, while the MSA generated by Praline obtained an SP score of 0.46, shift score of 0.28, and SPdist a score of 0.78.

To illustrate the importance of aligning residues approximately correct in terms of misplacement, as opposed to completely wrong, we show different alignments for reference set BB30009 as a case study (Fig 9). In this figure one can observe that this set consists of related protein subfamilies with well-defined core block regions, which are indicated by rainbow-colored columns. Furthermore, the subfamilies in this set also appear to be quite different in terms of sequence length.

In this case study we compared the query alignments generated by Clustal Omega and Praline because these two methods generated alignments with the largest contrast in terms of SP and SPdist scores for this set. Both query alignments obtained very similar SP scores: 0.48 for Clustal Omega and 0.46 for Praline. However, the difference in terms of avg_SPdist score between these query alignments is much more discernible, namely 0.59 for Clustal Omega and 0.78 for Praline. This outcome is highly interesting since one would not expect SP and avg_SPdist scoring to produce such different benchmark rankings. It is fair to stress, however, that this case study goes against the general trend in the observed benchmark tests, as Clustal Omega is shown to be the overall best performing MSA method in our benchmark.

A closer examination of reference and query alignments (Fig 9) reveals that Clustal Omega managed to get the intra-subfamilies alignment slightly better than Praline, but overall the Praline alignment contains more but less displaced mismatches. Since the SP scoring scheme treats every mismatch as equal, it is not surprising that the SP score for Clustal Omega alignment is better than the SP score for Praline alignment. However we can also observe from Fig 9 that Clustal Omega totally misaligns the subfamilies, whereas Praline aligns these more correctly. The relatively long inter-subfamily mismatches in the Clustal Omega alignment are appropriately punished in the SPdist scoring scheme resulting in the Praline alignment having a better avg_SPdist score.

This case study exemplifies that even though SP and SPdist scores are conceptually similar, the different treatment of mismatches may result in very different scores, which in turn may lead to different rankings in MSA benchmarking. Therefore, by additionally taking SPdist into account, one can get a more comprehensive assessment of the similarity between query and reference alignments. A thorough similarity comparison between two multiple sequence alignments is not only crucial in an MSA benchmark, but also plays a role in, for example, protein homology modeling: If one were to use the alignments of this case study to make a homology model, the Clustal Omega alignment would yield a considerably worse model as compared to a model obtained from the Praline alignment, since the latter alignment positions most of the conserved regions correctly or within relatively close proximity to each other.

Even though both the SPdist and shift scores take displacements of mismatches into account, the difference in the normalization procedures of these scores results in different behaviour between both scoring schemes. SPdist allows multiple mismatch shift thresholds, whereas the shift score only has a single constant (*ϵ*) which is used to differentiate between minor and major shift errors. The multiple mismatch thresholds in SPdist allow the user to determine the graveness of mismatch shifts in a more detailed way. S1 Fig shows that the SPdist score appears more expressive in the case of alignments of distantly related proteins, while the shift score shows more variance in the case of alignments of closely related sequences. In the benchmark case study of BAliBASE reference set BB30009 (Fig 9), where large mismatch shifts occur, the shift score appears unable to distinguish between the query alignments generated by Clustal Omega and Praline as both queries obtained shift score of 0.28.

#### SPdist analysis in alignment method optimization

**Fig 10:**
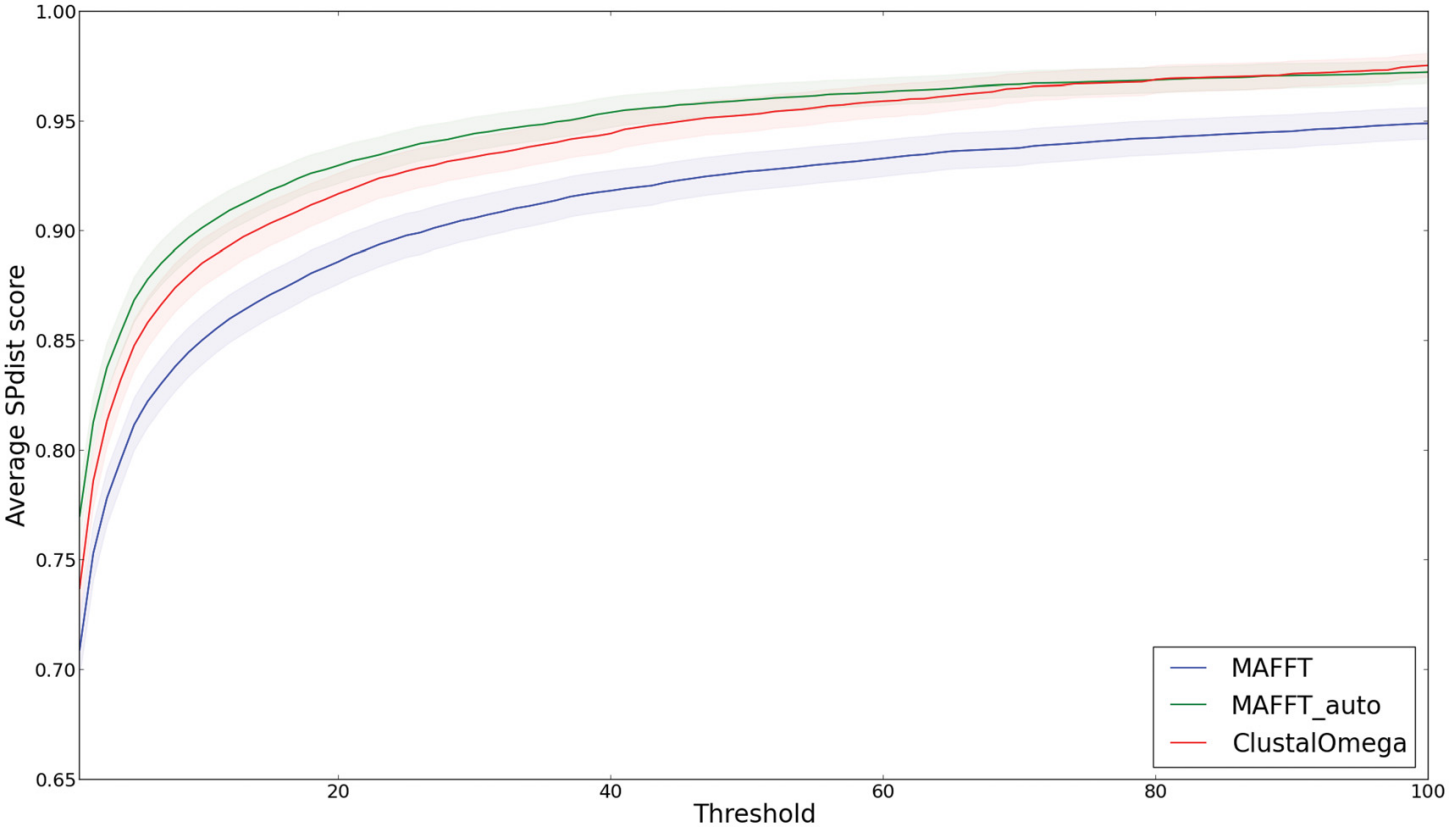
The average fraction of correctly aligned residues per alignment of all query alignments generated by Clustal Omega and MAFFT in the default and the “auto” setting. The shaded areas represent standard error ($SE=\frac{\sigma }{\sqrt{N}}$).

We compared the benchmark results of Clustal Omega and MAFFT, the latter of which was ran ran using its default (high-speed) and high-accuracy settings in order to illustrate how the SPdist scoring scheme can be employed to analyse the influence of parameter selection on alignment behaviour. The default setting of MAFFT is primarily aimed at aligning large datasets (where speed is often of the essence), whereas the “auto” setting aims to improve the accuracy by selecting the appropriate alignment optimization strategy which best suits the size of the input dataset. From Fig 10 one can observe a marked improvement on the quality of MAFFT alignments generated using the “auto” setting with respect to the alignments generated using the default setting. Interestingly, although MAFFT “auto” performs better at short distance thresholds, the convergence to a fully correct alignment at long distance thresholds appears to be slightly better for Clustal Omega. However the difference in terms of standard error is not significant. This shows that taking mismatch distance into account helps to obtain a richer performance comparison between the methods.

### Conclusion

Based on our observations, we argue that the severity of alignment shifts is often of relevance in numerous biological analyses, e.g. in homology modeling and manual alignment editing. It is therefore important to take the magnitude of mismatch displacement into account in MSA benchmark scoring procedures. Furthermore we showed that by using the SP score together with the SPdist score, one can discriminate the alignment quality better for alignments of distantly related sequences and/or difficult alignments (i.e. alignments that contains many gaps). Whilst classical scoring schemes such as SP scoring are useful for determining the overall similarity between reference and query alignments, scrutinizing the severity of mismatches as implemented in the SPdist score may provide a more salient similarity measure between the reference and the query alignments, particularly from the biological user perspective. The ability of observing detailed alignment quality is further enhanced by monitoring the behaviour of mismatch shifts over different distance thresholds (Figs 3, 4, and 10). For example, if an alignment column shows conserved Cysteine residues, a shift of a few positions can readily be corrected by a user, whereas longer shifts will lead the user to miss the overall conservation of the Cysteine. Therefore we recommend to use SP together with SPdist scoring in order to be able to measure to what extent a multiple sequence alignment actually is displaced with respect to the corresponding reference alignment. Understanding the behaviour of individual MSA methods in this respect provides a step towards optimized manual or semi-automatic MSA correction. Since MSA methods typically contain many adjustable parameters [37], this work provides a novel objective function that can be used when optimizing MSA methods on a reference alignment set.

## Supporting Information

S1 Fig Shift score and SPdist score compared to SP score. Here the reference sets 1–5 of the BAliBASE dataset are used to calculate the scores.
(EPS)

S1 Table Average SP, SPdist, and shift scores. The highest score for each category is written in bold. Notice that although SP and SPdist scores are conceptually similar to each other, they may lead to different rankings in an MSA benchmark. By observing the difference in rankings between bins with various cutoffs, one can see that some MSA methods are less prone to create large misalignment shifts than the others.
(XLS)
